# FID-Net: A versatile deep neural network architecture for NMR spectral reconstruction and virtual decoupling

**DOI:** 10.1007/s10858-021-00366-w

**Published:** 2021-04-19

**Authors:** Gogulan Karunanithy, D. Flemming Hansen

**Affiliations:** Department of Structural and Molecular Biology, Division of Biosciences, University College London, London, WC1E 6BT UK

**Keywords:** NMR, Deep learning, Non-uniform sampling, Virtual decoupling, Spectral reconstruction

## Abstract

In recent years, the transformative potential of deep neural networks (DNNs) for analysing and interpreting NMR data has clearly been recognised. However, most applications of DNNs in NMR to date either struggle to outperform existing methodologies or are limited in scope to a narrow range of data that closely resemble the data that the network was trained on. These limitations have prevented a widescale uptake of DNNs in NMR. Addressing this, we introduce FID-Net, a deep neural network architecture inspired by WaveNet, for performing analyses on time domain NMR data. We first demonstrate the effectiveness of this architecture in reconstructing non-uniformly sampled (NUS) biomolecular NMR spectra. It is shown that a single network is able to reconstruct a diverse range of 2D NUS spectra that have been obtained with arbitrary sampling schedules, with a range of sweep widths, and a variety of other acquisition parameters. The performance of the trained FID-Net in this case exceeds or matches existing methods currently used for the reconstruction of NUS NMR spectra. Secondly, we present a network based on the FID-Net architecture that can efficiently virtually decouple ^13^C_α_-^13^C_β_ couplings in HNCA protein NMR spectra in a single shot analysis, while at the same time leaving glycine residues unmodulated. The ability for these DNNs to work effectively in a wide range of scenarios, without retraining, paves the way for their widespread usage in analysing NMR data.

**Supplementary Information:**

The online version contains supplementary material available at 10.1007/s10858-021-00366-w.

## Introduction

Artificial intelligence (AI) and deep learning (DL) have led to huge advances in many fields, including computer vision and natural language processing, and is a methodology that is now embedded in many everyday technologies (Lecun et al. 2015). Unlike traditional methods in which a pre-defined algorithm for performing a task is provided, in deep learning, neural networks are trained to ‘learn’ a mapping between an input and a desired output. To achieve this, the network must first extract the relevant features from the input data to produce the required output. This flexibility has made deep learning particularly successful at performing tasks that are often intuitively straightforward for human beings to perform, but difficult to formalise into an algorithm (Goodfellow et al. 2016).

Over several decades, NMR researchers have sought to automate different aspects of NMR data analysis, speeding up the process and lowering the requirement for extensive training. However, many of these methods currently struggle to match human performance or can only do so in cases of data with sharp well-resolved peaks and minimal noise. Given its ability to outperform traditional methods at tasks that are intuitive for humans to perform, there is a huge potential for deep learning approaches to automate or improve different analysis stages within NMR spectroscopy, increasing the efficiency, utility, and ease of use of NMR. The current state of deep learning within NMR and potential future directions have been reviewed recently by Chen et al. (Chen et al. 2020).

An application of deep learning that has gained particular attention recently is the reconstruction of non-uniformly sampled (NUS) NMR spectra (Bostock and Nietlispach 2017; Miljenovic et al. 2018; Robson et al. 2019). NUS is an important tool for recording large multi-dimensional NMR datasets with high-resolution in a practical timeframe. In a uniformly sampled multidimensional NMR spectrum all points in the indirect dimensions on a Nyquist grid are sampled before the final frequency domain spectrum is obtained by a Fourier transform. Conversely, in a NUS spectrum only a subset of the points on the full Nyquist grid are collected. The points from the grid that are sampled are given by a sampling schedule and the percentage of sampled points from the full grid gives the sparsity. The task of a reconstruction algorithm is then traditionally to ‘fill in’ the missing points on the grid so that the reconstructed spectrum can be transformed with a discrete Fourier transform. The advantage of NUS is that it allows the spectroscopist to attain multidimensional spectra with many points and thus high resolution in a fraction of the time, though clearly this requires that the NUS spectrum can be reconstructed with high fidelity. With the advent of increasingly high-field NMR instruments, NUS is essential for exploiting the full-resolving power of these spectrometers, when both NMR time and sample stability can be limited. The development of new and better methods for reconstructing NUS spectra and for determining optimal sampling schedules thus remain active areas of research.

Several excellent algorithms for reconstructing NUS NMR spectra using non-DL methods exist including: SMILE (Ying et al. 2017), hmsIST (Hyberts et al. 2012) and MDD-NMR (Jaravine et al. 2006). Recent proof-of-principles studies have shown that DL based reconstruction methods have the ability to give reconstructions more rapidly and with higher fidelity than existing methods (Hansen 2019; Qu et al. 2020; Luo et al. 2020). Furthermore, these deep neural networks are trained and cross-validated exclusively on synthetic data, before being tested on real data. The ability to use synthetic data for training the neural networks overcomes a significant potential bottleneck of obtaining large amounts of curated data required for training.

Existing DNNs for reconstructing NUS data follow different strategies. Studies by Qu et al. and Luo et al. both reconstruct data from the frequency domain (Qu et al. 2020; Luo et al. 2020). In these cases, the main aim is to remove the aliasing artefacts caused by the non-uniform sampling. Qu et al. achieve this using an architecture composed of five stacked ‘dense-convolutional’ layers whereas Luo et al. employ stacked encoder-decoder blocks, sandwiched between convolutional layers from down and up sampling. On the other hand, we have previously shown that an architecture based on reconstructing the points in the time domain using a modified long short-term memory (LSTM) architecture is able to reconstruct lowly-sampled 2D spectra (12.5%), with lower error than either the SMILE or hmsIST algorithms (Hansen 2019).

Despite these successful applications of DL in NMR, all of the existing DL approaches for reconstruction still suffer from significant drawbacks. While they show good performance on spectra that strongly resemble their training data in terms of number of points, spectral width, sampling schedule and sparsity, they fail to match existing algorithms for reconstructions when the spectra deviate significantly from this. While the networks can in theory be retrained to reconstruct different spectra, the time and expertise required to do this likely represents an insurmountable hurdle to the widespread usage of such methods. Thus, an important factor that limits DNNs for NUS reconstructions relative to other currently existing methodologies is a lack of versatility.

We present below FID-Net: a versatile DNN architecture that is able to reconstruct the time domain of a diverse set of 2D NMR spectra with low error, matching or exceeding the performance of leading non-DL algorithms. FID-Net works effectively with arbitrary sampling schedules, meaning it can be deployed without further training and with minimal user input. We go onto demonstrate that this architecture is particularly adept at processing time domain data beyond reconstruction tasks. We show this network can also be used to virtually decouple ^13^C_α_-^13^C_β_ couplings in HNCA spectra in a single step, significantly improving the resolution of spectra.

## Results

### A flexible network architecture for analysing free induction decays

A key challenge when analysing free induction decays (FIDs; time domain data) is that the information about the individual resonances is contained within the entire length of the FID rather than within a localised section of it. This is unlike the frequency domain, where the information about a given resonance localises to a particular region in frequency space. From the perspective of designing DNNs for analysing time domain data it is necessary to find both long- and short-term patterns embedded within the whole sequence of data.

Recurrent neural networks (RNNs) have been successful architectures for analysing sequences. Within these networks each item in a sequence is fed through a series of cells and the output of cell *t* is a function of both input *t* and the output of cell *t*-1. In this way the network is able to keep a track of both the sequence history and current input to identify important features. Previously, we showed that a neural network based on a modified LSTM cells was capable of reconstructing 2D NUS data with low sampling (12.5%) with very high fidelity (Hansen 2019). The versatile FID-Net architecture demonstrated below is based on convolutional neural networks (CNNs) that connect both long- and short-term patterns to provide both high quality reconstructions and a high degree of flexibility.

Convolutional layers have been crucial in the success of DNNs for analysing images (Guo et al. 2016). They are based on the idea that input data often has a hierarchical structure and each layer in a CNN learns a filter with a user defined size. These filters are then convolved with the input from the previous layer such that they will be activated when certain features in the data are identified. Typically, filter sizes in convolutional layers are fairly small giving them a small receptive field i.e. they are only sensitive to localised features from an input and cannot ‘learn’ long-range patterns. While this is usually advantageous in image analysis, a large receptive field is required to extract information about resonances within an FID.

**Fig. 1:**
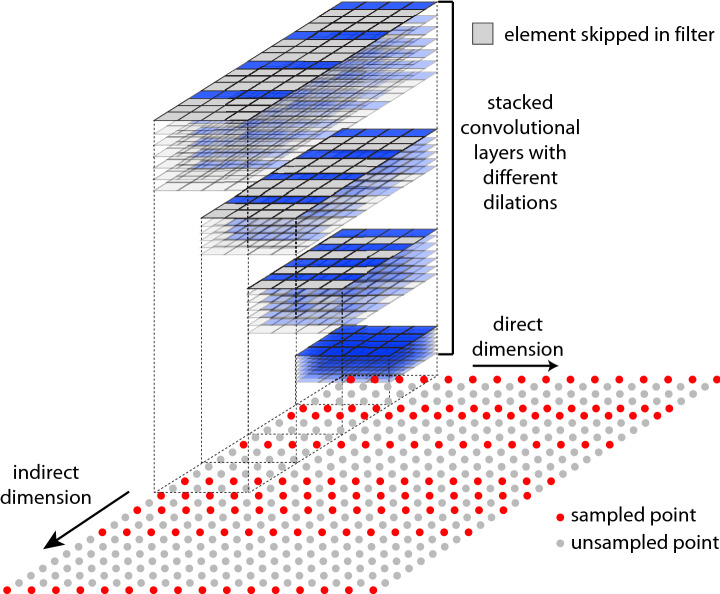
Schematic illustration of dilated convolutional layers that are extensively employed within FID-Net. These allow the network to have a large receptive field, required for analysing FIDs, whilst individual filters are able to remain relatively small in size

To overcome the receptive field issue of typical CNNs, FID-Net employs an approach similar to WaveNet, which was originally conceived in 2016 as a generative model for raw audio (Oord et al. 2016b). A raw audio signal is similar to an FID in that many time points are sampled in a short period of time and producing realistic audio requires an appreciation of both the short- and long-term patterns within the signal. In the WaveNet architecture, dilated convolution layers are used to give the network a large receptive field, which is capable of effectively analysing audio signals. Dilated convolutional layers skip a specified number of elements in the data, so it is effectively a convolutional layer with ‘holes’. By stacking convolutional layers with different dilation size, it is possible to create a block that acts like a normal convolutional layer with a very large filter size. This approach is indicated schematically in Fig. 1.

**Fig. 2:**
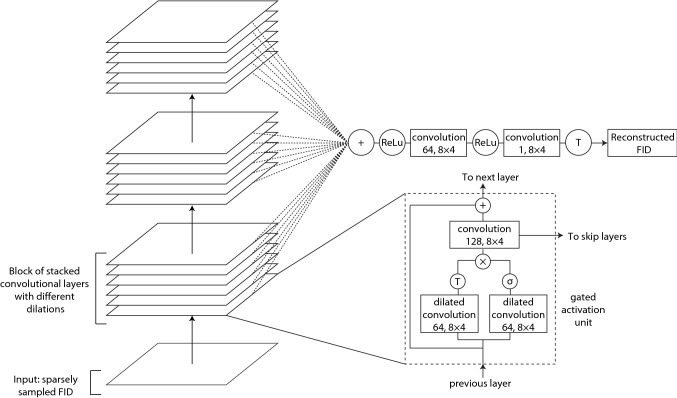
The full network architecture employed within FID-Net. The architecture is similar to WaveNet (Oord et al. 2016b), with main differences discussed in the text. The ‘ + ’ and ‘ × ’ symbols indicate the elementwise addition and multiplication operations. ‘ReLu’, ‘T’ and ‘σ’ symbols refer to rectified linear, hyperbolic tangent and sigmoidal activation functions respectively

In FID-Net, the approach of dilated convolutions along with other features from WaveNet are employed. Gated activation units, as previously employed in the PixelCNN (Oord et al. 2016a) are used: these have both hyperbolic tangent and sigmodal activation functions in individual convolutional layers to help model the complex signal. FID-Net also employs skip and residual connections to aid the training and speed of convergence (He et al. 2015). However, a number of significant differences between the WaveNet and FID-Net architectures also exist. Firstly, the WaveNet architecture was designed as a generative audio model. Therefore, it is important that the temporal ordering of data points is maintained and predicted outputs depend only on preceding values. Consequently, causal padding is used in the convolutional layers. Conversely, in the FID-Net model the aim is to recapitulate the full FID, for example, from a sparse starting point. To achieve this the FID-Net architecture needs to look both backward and forwards to help ascertain the correct value of a given FID time point. Secondly, in the WaveNet model a kernel size of two was used, whereas in FID-Net this is increased to eight. This is important when dealing with sparse data as a small kernel size will result in most inputs to the convolutional layers having no information. With a kernel size of eight, when dealing with a sampling rate of 12.5% in a 2D NMR spectrum, each time the convolutional filter is applied one of the input values will on average be non-zero. Thirdly, the WaveNet model considers a single audio input channel at a time. In the context of NMR data, adjacent indirect FIDs will share features, for example, when analysing 2D spectra the direct dimension is first Fourier transformed and a resonance in such a spectrum, (ω_2_,*t*_1_), spans several ω_2_ slices. Therefore, in FID-Net 2D convolutions are used and four FIDs are simultaneously reconstructed, giving an overall kernel size of 8 × 4. The model is applied as a sliding window across the input data to yield the final output. The full FID-Net architecture is given in Fig. 2.

### Reconstructing 2D NUS spectra

**Fig. 3:**
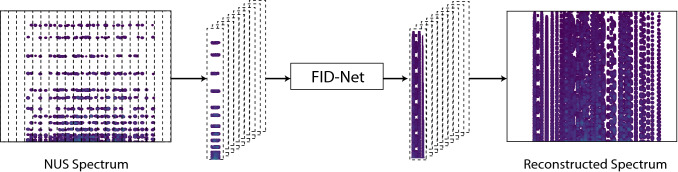
Schematic illustration of how reconstructions of 2D NUS spectra are performed by FID-Net. The NUS spectrum is Fourier transformed in the direct dimension and then segmented. The segmented sections form a batch that is then reconstructed in parallel and stitched back together to yield the final reconstructed spectrum

In its current form, FID-Net is only able to reconstruct four FIDs at a time. However, the neural network is applied as a sliding window across the 2D NUS spectrum, Fig. 3, so that each individual FID is analysed four times and the average taken to yield the final reconstructed or otherwise transformed spectrum. To speed up the reconstructions the ability of modern GPUs is leveraged to perform a very large number of calculations in parallel and batch the input FIDs into the appropriate number of segments containing four points in the direct dimension. The segmented FIDs are then all transformed in parallel and the fully reconstructed spectra can be stitched back together as indicated in Fig. 3. Using this approach on an Nvidia 1070 Ti GPU card, 2D spectra can be reconstructed in a few seconds, a similar timeframe to either the SMILE or hmsIST algorithms. The latter algorithms do not require the use of a GPU for rapid performance, but these cards are now relatively affordable (the Nvidia 1070 Ti GPU used here is now available for less than $500) and users can use freely available resources on NMRBOX (Maciejewski et al. 2017) for the processing of NMR spectra.

As detailed in the methods (see below) the FID-Net architecture is trained on a diverse training set. This includes spectra with varying numbers of complex points, sweep widths and for each pass through the neural network a new random sampling schedule is calculated and applied to the spectrum and noise is added to the input sparsely sampled spectrum. This endows FID-Net with a high degree of robustness and flexibility compared to other DL approaches for spectral reconstruction.

In a very recent and elegant study, it was shown that a neural network could be interpreted and thus it was shown how each of the hidden layers can be mapped to specific actions and mathematical transformations (Amey et al. 2021). Such an approach is naturally highly attractive in order to fully understand the strengths and weaknesses of a DNN. However, with the large size of FID-Net, both in terms of number of hidden layers and number of trainable parameters, focus below is on evaluating the robustness and the versatility of FID-Net for reconstruction of NUS spectra as well as estimating when the network performs well, as opposed to delineating each of the transformations within the network.

To rigorously test the ability of the FID-Net architecture to reconstruct NMR spectra with high fidelity with respect to important spectroscopic parameters (linearity of recovered peak intensity, accuracy of recovered frequencies, recovery rate of actual peaks and minimisation of false positives) we use a small synthetic spectrum composed of nine partially overlapping peaks with different intensities (see supporting material and Fig S1). In this synthetic spectrum the ground truth parameters for the peaks can be accurately obtained and therefore robustly compared to those obtained for the reconstructions. The reconstructions are also carried out using the SMILE and hmsIST algorithms for comparison. This analysis reveals that FID-Net is particularly successful at reconstructing spectra at very low sparsity and in the presence of appreciable noise compared to the other algorithms (Fig. S2–S6). The DNN strikes a favourable balance between recovering real peaks with high probability while minimising the presence of artefacts even for challenging situations. When dealing with data at a higher sampling rate than the DNN is trained at, FID-Net maintains a high level of performance but the linearity of recovered peak intensities and accuracy of recovered frequencies is less than can be achieved using SMILE or hmsIST. However, the performance of these well-established algorithms in the presence of noise drops much more than FID-Net and the lack of false positives or false negatives when using FID-Net is a clear advantage.

To demonstrate the versatility of the FID-Net architecture in the context of biomolecular NMR the network is used to reconstruct a varied set of typical biomolecular NMR spectra. The results of reconstructing spectra on three different proteins are again compared to reconstructions obtained using the SMILE and hmsIST algorithms. Specifically, the spectra reconstructed are a ^15^N-^1^H HSQC spectrum of T4 Lysozyme (19 kDa) with a large sweep width in the indirect dimension (Hansen 2019), a ^15^N-^1^H HSQC spectrum of the SH3 domain from ABP1P (6.5 kDa), and a ^13^C-^1^H methyl-TROSY HMQC spectrum of HDAC8 (42 kDa). In all cases the reconstructions are conducted with 12.5% sampling and reconstructions are performed 200 times per spectrum with different sampling schedules: 100 sampling schedules with a different Poisson-gap sampling schedule (Hyberts et al. 2010), with sinusoidal 0 → *π*/2 weighting that biases towards early time points, as well as 100 different random sampling schedules. The parameters in each of the spectra are summarised in Table 1. Given the very low sparsity of the data and the large number of signals compared to the number of sampled points these reconstructions (particularly for T4 Lysozyme and HDAC8) are very challenging.

**Table 1:** Summary of spectral parameters of the spectra employed in benchmarking FID-net

Protein	Spectrum type	B_0_ (MHz)	# complex points	Indirect sweep width (Hz)	Half-dwell 1st point
SH3 domain	^15^N-^1^H HSQC	600	120	1800	Yes
T4 Lysozyme	^15^N-^1^H HSQC	700	256	5100	No
HDAC8	^13^C-^1^H HMQC	800	192	4500	Yes

**Fig. 4:**
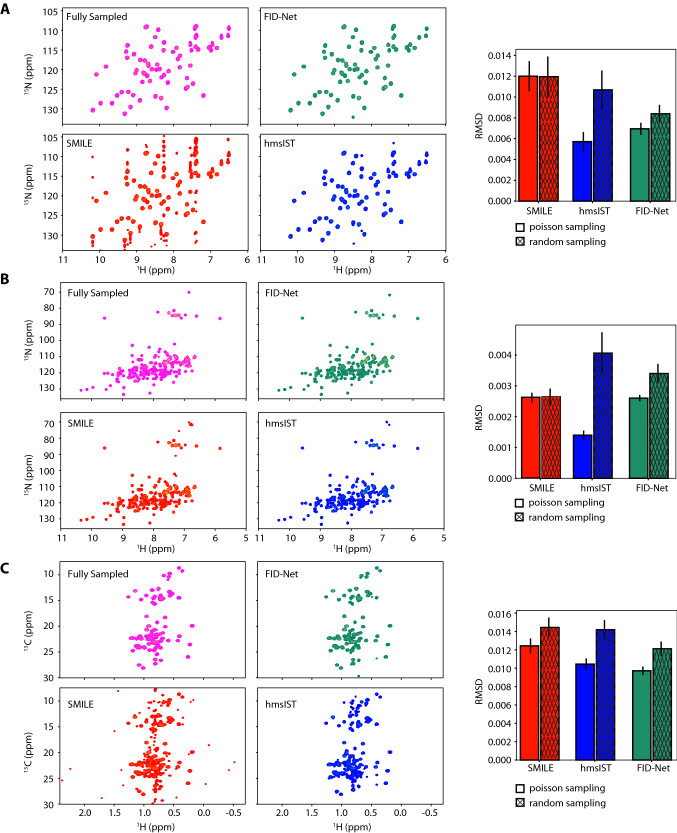
Exemplar reconstructions and algorithm performance for (**a**) the SH3 domain from ABP1P, (**b**) T4 Lysozyme. and (**c**) human histone deacetylate 8 (HDAC8). Spectral parameters are listed in Table 1. In all cases the exemplar spectra are normalised and plotted at the same contour level and only positive contours are shown. Exemplar reconstructions are performed from the same Poisson-gap sampling scheme with 12.5% sampling, which are listed in the supporting material (Table S1). The bar graphs indicate the average and root-mean-squared-deviation (RMSD) between the normalised reconstructed and fully sampled spectra using 100 Poisson-gap sampling schedules and 100 random sampling schedules

The reconstruction results are shown in Fig. 4. In general, it is clear that all three methods, SMILE, hmsIST, and FID-Net, can provide good quality reconstructions, even at the low sampling rate (12.5%) considered here. For the hmsIST algorithm, performance improves substantially using Poisson-gap sampling schedules over random sampling, which has been shown previously (Hyberts et al. 2012). In contrast, for SMILE there is generally minimal advantage to using Poisson-gap sampling schedules. Using FID-Net there is an advantage to using Poisson-gap sampling schedules, but this is less marked than with hmsIST and the performance with random sampling schedules is also generally good, highlighting the robustness of the neural network approach.

While the performance between the algorithms is not too dissimilar, the ability of a single FID-Net to reconstruct a diverse set of spectra, including very challenging cases with a large number of signals compared to sampled points, with high quality and without retraining, represents a clear advance compared to previous neural networks. Another significant benefit of the FID-Net is that it can be run with minimal user input, since, like other DL methods, it contains no user adjustable parameters. All that is required is that the data is phased and Fourier transformed in the direct dimension and that the sampling schedule is provided so that the non-sampled points can be filled with zeros prior to reconstruction. This relative ease of use facilitates that FID-Net can be easily implemented into automated data-processing pipelines incorporating NUS data.

**Fig. 5:**
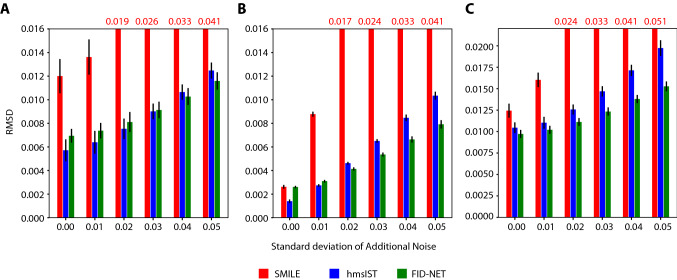
Variation in reconstruction quality for the different methods as a function of added Gaussian noise with standard deviation between 0.01 and 0.05 for **a** SH3 domain from ABP1P, **b** T4 Lysozyme and **c** human histone deacetylate 8 (HDAC8). In all cases the 12.5% sampling is used with 100 different Poisson-gap sampling schemes. The bars indicate the average normalised RMSD of the 100 reconstructions between the reconstructed spectra and fully sampled spectrum with no added noise. The values in red indicate the RMSD for SMILE reconstructions when these are not within the y-axis range

A further advantage of using FID-Net is its robustness. To test this, we add different amounts of additive Gaussian noise to our benchmark spectra prior to performing the reconstructions. Algorithms such as hmsIST and SMILE can through multiple iterations converge on very accurate reconstructions in the presence of little noise as is the case in the benchmark spectra. A key advantage of the DNN approach is its ability to identify underlying resonance frequencies even in noisy data. This is demonstrated in Fig. 5: while FID-Net does not always give the highest fidelity reconstructions in the absence of additional noise, the reconstruction quality is maintained better than SMILE or hmsIST in the presence of noise (exemplar reconstructions with added noise are shown in the supplementary material Fig. S7-9). As alluded to above with the synthetic spectrum, FID-Net also shows good robustness with respect to the sampling rate in the NUS data, even when this is far from the sampling rate employed in training (Wang et al. 2020) (Fig. S10).

It should be noted that in the above analyses we have chosen the normalised RMSD between the reconstructed and fully sampled spectra as the metric for comparing the different reconstruction methods. This is a simple and robust method that penalises missing or lower intensity peaks as well as spectral artefacts or higher intensity peaks equally. Given that the dynamic range of the peaks in the benchmark spectra considered here is not large, the calculation is not dominated by a small number of high intensity peaks. For reconstructions performed with SMILE, the intensity of the reconstructed peaks is down-scaled to avoid noise spikes appearing as false-positive peaks. Consequently, the normalised RMSD becomes very high in the presence of noisy data (Fig. 5). The parameters we have employed here for the SMILE reconstructions give generally good results over a range of sampling schedules, however, the parameters used in SMILE reconstructions may be improved on a case-by-case basis to improve the quality of reconstructions. Conversely, with DL methods such as FID-Net no such optimisation is required by the user to achieve optimal results.

One issue that exists with all existing methods for performing reconstructions is that they do not provide a direct measure of confidence in their final outputs, although attempts have been made to estimate the confidence using statistical methods such as the delete-d jackknife procedure (Mayzel et al. 2017). Deep neural networks provide a relatively easily method for giving a measure of confidence by training and deploying an ensemble of networks and measuring the variation in their outputs, as was done in the deep neural network processing of DEER data (Worswick et al. 2018). The relatively long training times required to train an FID-Net preclude training a large ensemble of networks, but the fact that FID-Net is applied as a sliding window with four FIDs reconstructed simultaneously can be used to provide insight into the confidence in the output. Each slice in the reconstructed spectrum is reconstructed four times (at a different point in the kernel in the direct dimension). The final reconstruction is found by taking the average of these four reconstructions. A measure of confidence in the final result can therefore be provided by considering the standard deviation of the four individual reconstructions. The results of this analysis for the SH3 domain protein, T4 Lysozyme and HDAC8 proteins are shown in Fig. S11.

### Virtual decoupling of HNCA spectra

FID-Net is a versatile architecture capable of being trained to perform tasks beyond reconstruction. Below, the FID-Net architecture is trained to perform the task of virtually decoupling ^13^C_α_-^13^C_β_ couplings in triple-resonance HNCA and HN(CO)CA spectra (Kay et al. 1990). HNCA and HN(CO)CA are amongst the most important three-dimensional spectra in biomolecular NMR for obtaining backbone ^1^H, ^15^N and ^13^C_α_ chemical shift assignments (Kay et al. 1990). Unfortunately, even when using long acquisition times in the ^13^C dimension, the resolution of the ^13^C_α_ peaks is limited by the one-bond scalar coupling between ^13^C_α_ and ^13^C_β_ nuclei. This coupling, of approximately 35 Hz and substantially larger than the typical intrinsic linewidth of ^13^C_α_, is present for all residues except for glycine. Successfully removing the coupling from spectra improves both their sensitivity and resolution, aiding the assignment procedure. This is particularly important for large and/or intrinsically disordered proteins where signal overlap becomes more severe.

A number of approaches have been attempted to experimentally eliminate the ^13^C_α_-^13^C_β_ couplings in HNCA and HN(CO)CA spectra. However, none of these have been completely successful and complications include glycine residues, which do not have a ^13^C_α_-^13^C_β_ coupling as well as serine and threonine residues, where the ^13^C_β_ chemical shifts overlap with the ^13^C_α_ region. An alternative to experimental decoupling is to record a conventional HNCA or HN(CO)CA experiment, potentially with NUS, and then virtually decouple it in a post-acquisition step. A number of methods have already been developed to perform virtual decoupling using deconvolution followed by maximum entropy reconstruction (Shimba et al. 2003; Delsuc and Levy 1988) and in a recent study by deconvolution with compressed sensing reconstruction (Kazimierczuk et al. 2020). With both of these methods care must be taken with regards to glycine residues that do not form doublets in the ^13^C dimension. In the previous methods, a single average *J*-coupling constant must also be assumed for the entire spectrum, which may lead to distortions in the spectrum if the spectral linewidth is less than the difference between the actual and average coupling constant.

**Fig. 6:**
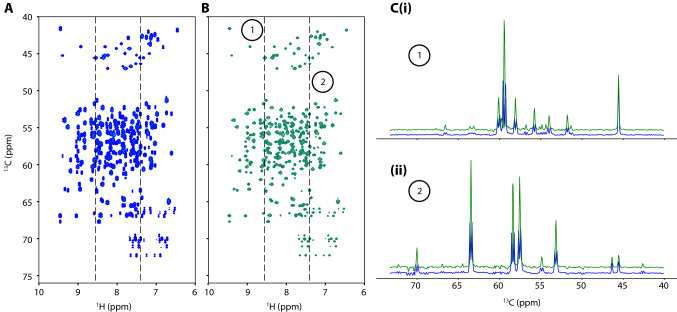
^1^H-^13^C projection from an HNCA spectrum of T4 Lysozyme **a** before (blue) and **b** after (green) virtual decoupling. Only positive contours are shown. The 1D ^13^C slices taken from the spectra in panels **c** (i) and (ii) demonstrate how the doublets in the spectrum are successfully decoupled yielding an improvement in resolution and two-fold increase in sensitivity for these peaks while the singlet glycine peaks are unaltered

By contrast, as shown below, FID-Net can be trained to virtually decouple the ^13^C_α_-^13^C_β_ doublets in a single step and with no user intervention. Figure 6 shows the application of a FID-Net to virtually decouple an HNCA spectrum of T4 Lysozyme. Essentially, all non-glycine residues are successfully decoupled giving a factor of two increase in the sensitivity and significantly improving the spectral resolution, while the glycine residues are left unaltered. For both decoupled and unaltered (glycine) residues the line shape following the virtual decoupling is excellent and shows no evidence of artefacts.

The FID-Net for virtual decoupling has no user defined parameters and so can easily be applied as part of automated or semi-automated processing routines. It is trained to run on fully sampled data and is a separate network with different parameters to the FID-Net described above for reconstruction, although the same architecture. The network can be applied to a dataset that has been experimentally fully acquired (as is done here) or to a non-uniformly sampled dataset that is first reconstructed, either using another FID-Net or another algorithm. By combining virtual decoupling with NUS it is possible to dramatically reduce the time required to attain highly resolved HNCA and HN(CO)CA spectra, reducing the overall time requirement for backbone assignment.

## Methods

### Network architecture

The FID-Net architecture consists of many convolutional layers stacked into residual units, as shown in Fig. 2. Each residual unit consists firstly of a dilated convolutional layer composed of n filters (n = 128 for reconstruction network and n = 64 for decoupling network) with an 8 × 4 kernel size. Half of the filters are activated by a sigmoidal activation function and the other half by a hyperbolic tangent function. The results of these two activations are then multiplied (creating a gated activation unit) and passed through a second convolutional layer, with no dilation, also composed of n filters and an 8 × 4 kernel size. The output of this layer is passed to the end layers of the network and also added to the input of the layer to create the input for the next gated activation unit in the network.

The dilations rates employed for FID-Net are cycled through the values: 1, 2, 4, 6, 8, 10, 12, 14, 16, 20, 24, 28 and 32. These dilations rates were empirically found to give good performance at both reconstructing and virtual decoupling. Given the time taken to the train the network (on the order of weeks) a detailed comparison of the effects of different dilations rates has not been possible. These dilation rates are cycled through three times to make the full FID-Net, as indicated by the three blocks in Fig. 2.

The outputs of all the individual gated activation units are then summed and passed through a further convolutional layer with rectified linear activation. The final output is produced with a final convolutional layer composed of a single filter followed by hyperbolic tangent activation to ensure the values are between − 1 and 1. The python code used to create the neural network is provided in the supplementary materials.

The total number of trainable parameters in the reconstruction network is 30,424,897 and 7,610,273 for the decoupling network, reflecting the increased number of filters in the reconstruction network. While the increased number of filters in the reconstruction network improves its performance, excellent performance for the decoupling network is achieved with a smaller network that has the benefit of being faster to train (vide infra).

### Training

In common with other recent DL networks for reconstructing non-uniformly sampled data, FID-Net is trained exclusively on synthetic data. The synthetic data is generated using the equation:

$$S\left({t}_{1}, {t}_{2}\right)=\sum_{n}{A}_{n}exp\left(-i2\pi {\nu }_{1,n}{t}_{1}\right)\mathrm{exp}\left(-{R}_{2}^{\left(1,n\right)} {t}_{1}\right)\mathrm{cos}(\pi {J}_{n}{t}_{2})exp(-i2\pi {\nu }_{2,n} {t}_{2})\mathrm{exp}(-{R}_{2}^{(2,n)}{t}_{2})$$

where *n* runs over the number of signals in the plane, *A*_*n*_ is the amplitude of signal *n*, *ν*_1,n_ and *ν*_2,n_ are the frequencies of signal *n* in the direct and indirect dimensions respectively, ${R}_{2}^{\left(1,n\right)}$ and ${R}_{2}^{\left(2,n\right)}$ are the transverse relaxation rates in the direct and indirect dimensions respectively for signal *n* and *J*_n_ is the *J* coupling constant for signal *n*. The times *t*_1_ and *t*_2_ are given by multiplying 1/SW (for the relevant sweep width) by the series 0, 1, …*N*-1 where *N* is the number of complex points in this dimension.

For the reconstruction network: For each plane in the training set parameters are randomly (uniformly) selected from the intervals listed in the table below:

**Table:** 

Number of signals	50–250
Amplitude^a^	0–2.0
Direct dimension complex points	128–512
Indirect dimension complex points	100–256
Direct dimension SW (Hz)	1500–3000
Indirect dimension SW (Hz)	1800–5400
J (Hz)	0.0
${R}_{2}^{\left(1\right)}$(s^−1^)	5.0–60.0
${R}_{2}^{\left(2\right)}$(s^−1^)	5.0–60.0

^a^Normal distribution with mean 1.0 and SD 0.5 that is truncated to between 0.0–2.0

For the virtual decoupling network: For each plane in the training set parameters are randomly (uniformly) selected from the intervals listed in the table below:

**Table:** 

Number of signals	10–70
Amplitude^a^	0–2.0
Direct dimension complex points	128–512
Indirect dimension complex points	192–256
Direct dimension SW (Hz)	1800–5400
Indirect dimension SW (Hz)	4000–8000
J (Hz)	28–40^b^
${R}_{2}^{\left(1\right)}$(s^−1^)	5.0–60.0
${R}_{2}^{\left(2\right)}$(s^−1^)	5.0–60.0

^a^Normal distribution with mean 1.0 and SD 0.5 that is truncated to between 0.0 and 2.0.

^b^In training for 12% of residues couplings are randomly set to 0 Hz to reflect the presence of glycine residues.

For both networks, frequencies ν_1_ and ν_2_ are determined by generating a random number in the interval [− 0.5, 0.5] and multiplying this by the relevant sweep width for the plane.

To simulate the reconstruction process, the calculated 2D FIDs are phased and Fourier transformed in the direct dimension and random Gaussian noise with standard deviation between 0.001 and 0.03 is added to the input plane. Four consecutive points in the plane are then randomly chosen for each member of a training batch. A random sampling schedule is calculated on the fly at a given sampling level (this is always set to 12.5% for applications here and the first point is always selected) and applied to the plane to give a non-uniformed sampled section. This means that in each epoch of training, the model will be trained on a different part of each plane subjected to a different sampling schedule. This substantially increases the effective size of the training data and minimises the potential for overfitting. The real and imaginary components of each complex point in the indirect NUS dimension are interleaved. To ensure the size of individual tensors in each batch are uniform they are zero filled up to 512 points so that each input plane in the batch has 512 × 4 points. The planes are normalised according to the highest intensity point.

The training data for virtual decoupling is very similar except for an average of 88% of residues in each plane also have a *J*-coupling of 28–40 Hz in size to match the size and spread of protein ^13^C_α_-^13^C_β_ couplings. The 12% of peaks with no coupling mimic glycine residues. The number of resonances in each plane is also reduced to between 10 and 70, reflecting the increased sparsity of three-dimensional spectra.

The DL models are developed and trained using the Tensorflow library (Abadi et al. 2016) with the Keras front-end (Chollet 2015). The cost function used to optimise the reconstruction network was the mean squared error between the fully sampled data (with no noise) and the reconstructed data in the frequency domain. The cost function for the virtual decoupling network was the mean squared error between the virtually decoupled spectrum produced by the network and the fully decoupled synthetic spectra (with no noise) in the frequency domain. The RMSprop optimiser (Hinton 2012) is used for training. Initially the learning rate was set to 10^–4^ until the change in validation loss between epochs plateaus. The learning rate was then reduced to 10^–5^ and continues until the validation loss plateaus. For the reconstruction FID-Net, the RMSD between the fully sampled and reconstructed FIDs was 0.0278 at the end of training for the model used here. A batch size of 16 was used. On a standard desktop computer (Intel i7-7700 CPU @ 3.6 GHz and 16 GB RAM) equipped with an Nvidia Geforce GTX 1070 GPU the total training time was approximately two weeks for the reconstruction network. The final RMSD for the virtual-decoupling FID-Net was 0.0017. The decoupling network was trained on a desktop computer with the same specifications and the total training time for the network was approximately one week (the faster training time reflecting the smaller network).

For the application of FID-Nets for the reconstruction and virtual decoupling of actual spectra, extensive use is made of the nmrGlue python module for the reading, writing and manipulation of spectra (Helmus and Jaroniec 2013). In these cases, synthetic NUS data at a given sampling level are produced from the fully sampled data by removing points in the indirect dimension. The performance of the algorithms is then compared by calculating the RMSD with respect to the fully sampled spectrum.

### NMR spectroscopy

T4 Lysozyme: A 2D ^15^N-^1^H HSQC correlation spectrum was recorded on a uniformly ^13^C, ^15^N isotope labelled sample of the L99A mutant of T4 Lysozyme. The sample was prepared as described previously (Bouvignies et al. 2011). The NMR spectrum was recorded at 298 K on an NMR spectrometer operating at 700 MHz ^1^H Larmor frequency and equipped with a helium-cooled TCI inverse cryoprobe. The fully sampled spectrum was acquired as a 1024 × 256 complex matrix with spectral widths of 12 kHz (^1^H) and 5.1 kHz (^15^N). An adiabatic ^13^C inversion pulse was applied in the centre of the ^15^N chemical shift evolution period to refocus ^15^N-^13^C_α_ and ^15^N-^13^CO scalar couplings. Four scans were collected for each t_1_ increment with a recycle delay of 1 s giving a total experiment time of 34 min.

The 3D HNCA spectrum was recorded on a triple labelled (^2^H, ^13^C, ^15^N) sample of T4 Lysozyme at 298 K. The spectrum was recorded on an NMR spectrometer operating at 600 MHz ^1^H Larmor frequency equipped with a room temperature TXI HCN inverse probe. The fully sampled spectrum was acquired as a 1024 × 28 × 256 complex matrix with spectral widths of 10.8 kHz (^1^H), 1.8 kHz (^15^N) and 5 kHz (^13^C). ^2^H decoupling is applied through the sequence to eliminate scalar couplings to these nuclei and selective shaped inversion pulses are employed to remove couplings to ^13^CO nuclei. Eight scans were collected for each increment with a recycle delay of 1.5 s giving a total experiment time of approximately 112 h.

SH3 domain of ABP1P: A 2D ^15^ N-^1^H HSQC correlation spectrum was recorded on a uniformly ^13^C, ^15^N isotope labelled sample of the SH3 domain of ABP1P, prepared as described previously (Vallurupalli et al. 2007). The spectrum was recorded at 298 K on an NMR spectrometer operating at 600 MHz ^1^H Larmor frequency and equipped with a room temperature TXI HCN inverse probe. The fully sampled spectrum was acquired as a 513 × 120 complex matrix with spectral widths of 8 kHz (^1^H) and 1.8 kHz (^15^N). A composite ^13^C inversion pulse was used in the centre of the ^15^N chemical shift evolution period to refocus scalar couplings. Eight scans were collected for each t_1_ increment with a recycle delay of 1 s giving a total experiment time of 36 min.

HDAC8: A 2D ^13^C-^1^H HMQC methyl-TROSY correlation spectrum was recorded on a methyl-labelled (isoleucine, leucine and valine methyl groups are isotopically ^13^C-^1^H labelled with specific pro-(S) labelling for leucine and valine groups) HDAC8 sample, prepared according to previously reported protocols (Werbeck et al. 2020). The spectrum was recorded at 298 K on an NMR spectrometer operating at 800 MHz ^1^H Larmor frequency and equipped with a helium-cooled TCI inverse cryoprobe. The fully sampled spectrum was acquired as a 1024 × 192 complex matrix with spectral widths of 12.5 kHz (^1^H) and 4.5 kHz (^13^C). Four scans were collected for each t_1_ increment with a recycle delay of 1 s giving a total experiment time of approximately 28 min.

The spectra are phased and Fourier transformed, with the relevant regions subsequently extracted using NMRPipe (Delaglio et al. 1995).

### Reconstructions of NMR spectra

Reconstructions using the hmsIST algorithm were performed using the default parameters. For reconstructions with SMILE the default parameters are employed except that the -nsigma parameter is set to 3; in our hands, using a higher value results in poorer reconstructions for the spectra considered here. The Fourier transform and phasing parameters are set as appropriate for each spectrum and a squared sine-bell window function is used (− xQ1 0.42 − xQ2 0.98 − xQ3 2.0). The hmsIST and FID-Net reconstructed spectra are multiplied by the same window function and all spectra Fourier transformed in the indirect dimension. For the RMSD calculations all spectra are normalised according to the maximum intensity point.

### Virtual decoupling of HNCA spectra

For virtual decoupling of 3D HNCA spectra, the spectrum is first processed and Fourier transformed in the ^1^H and ^15^N dimensions and kept in the time domain in the ^13^C domain. Each ^1^H-^13^C plane is then decoupled sequentially with the network passed as a sliding window over the ^1^H points as is done for the reconstruction network. After each plane has been decoupled the points in the ^15^N dimension are recombined to give a 3D HNCA with the ^13^C plane in the time domain. This ^13^C dimension is then processed and Fourier transformed to yield the final pure frequency-domain spectrum. Figure 6 shows the ^1^H-^13^C projection of the 3D spectrum.

## Conclusion

We demonstrated a new versatile deep learning architecture, FID-Net, that can be trained to perform several tasks within common biomolecular NMR spectroscopy, including reconstruction and virtual decoupling of spectra. A key strength of the networks presented is their robustness and ability to work effectively in a wide range of scenarios without a requirement for further retraining and no user adjustable parameters. This flexibility paves the way for these analyses to be incorporated as part of automated or semi-automated processing schemes and the use of deep learning analyses within the NMR community more generally. Moreover, reconstruction and virtual decoupling with FID-Net also provide the confidence level of the performed transformations, something that is unique to the presented method compared with other tools for reconstruction. As demonstrated with the virtual decoupling, DL approaches can also be trained to perform tasks that are difficult for traditional algorithmic approaches without prior assumptions, opening up new opportunities to perform innovative analyses with NMR data.

## Supplementary Information

Below is the link to the electronic supplementary material.

Supplementary file 1 (PDF 3968 kb)

## Data Availability

The code (python) used for reconstruction and virtual decoupling is available from the corresponding author upon reasonable request. Code for reconstruction and virtual decoupling using FID-Net is available on GitHub: https://github.com/gogulan-k/FID-Net.
