# Supplementary material for: FID-Net: A versatile deep neural network architecture for NMR spectral reconstruction and virtual decoupling
Source: J Biomol NMR. 2021 Apr 19;75(4):179–91. doi: 10.1007/s10858-021-00366-w (PMC8131344; doi:10.1007/s10858-021-00366-w)
Supplement: Supplementary file 1 — (PDF 3968 kb) [file 10858_2021_366_MOESM1_ESM.pdf]

Supporting Material for

FID-Net: A Versatile Deep Neural Network Architecture for Spectral Reconstruction and Virtual Decoupling

Gogulan Karunanithy and D Flemming Hansen

Reconstructing Synthetic Data

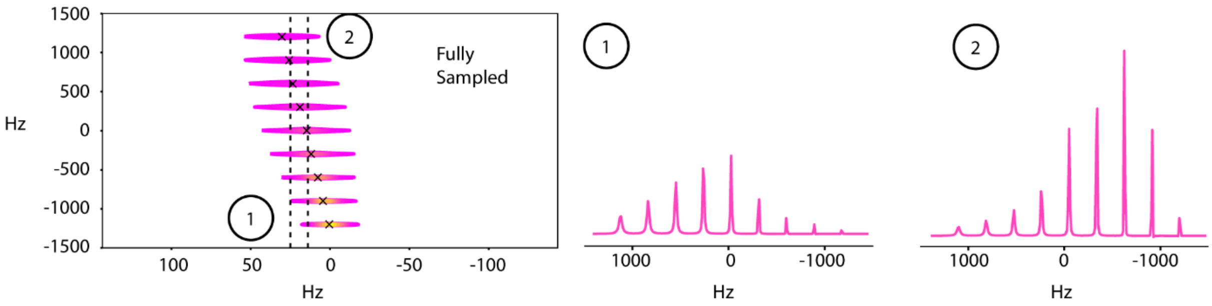

Fig S1 Synthetic NMR spectrum used to assess the quality of FID-Net reconstructions with NUSCON metrics. Crosses indicate the peak centres.

In order to robustly test the performance of the FID-Net reconstruction algorithm using NUSCON metrics (https://nuscon.org/metrics.pdf) we create a synthetic NMR spectrum shown in Fig S1. This allows us to robustly determine the ground truth values of peak parameters that can then be compared to the reconstructed spectra. The spectrum consists of nine slightly offset peaks that gradually decrease in intensity and increase in linewidth. The largest peak has an intensity of 1 and the smallest 0.04: the relatively large dynamic range here makes RMSD a less optimal tool for determining the quality of reconstruction since this will be dominated by the very intense peaks. The vertical slices of the spectrum that are reconstructed below consist of 256 complex points. Given that each vertical slice can consist of up to nine peaks this makes the reconstructions challenging at the sampling rates considered here given the large number of peaks compared to the number of sampled points.

In this assessment, a master peak list is first generated from the fully sampled synthetic data (Fig S1). For each condition (given sampling rate and noise) we then generate 100 non-uniformly sampled datasets with different Poisson-gap sampling schedules. The NUS spectra are then reconstructed using FID-Net as well as SMILE[1] and hmsIST[2] for comparison and a peak picker is then applied to the reconstructed spectra for comparison to the original. The use of Poisson-gap sampling schedules here does not disadvantage SMILE, which works equally well with random and Poisson-gap sampling schedule, but it necessary for optimal performance using hmsIST. After reconstruction the *rescale.com* tool within *NMRPipe*[3] is applied to all reconstructed spectra so that their maximum intensity is 1.0. The *NMRPipe* peak picker is used for determining the existence and properties of peaks and the same parameters are used for all reconstruction tools. The following criteria are used to assess the fidelity of reconstructions:

1. Linearity of reconstructed intensities: here this is done by measuring the intensity of peaks in the reconstructed spectrum and comparing these to do the intensity of peaks in the fully sampled spectrum by calculating the R^2, coefficient of determination. The R^2 calculation assumes there should be a perfectly linear relationship between the intensities of the recovered and master peaks, *i.e.*, we fit to the line $y = x$. Note the values here will in general be different to the square of the correlation coefficient that assumes a model of the form $y = ax + b$ for some numbers a and b with $a \neq 0$. Only where there is a clear correspondence between a master and recovered peak is this included in the calculation: for each master peak the corresponding recovered peak (if this exists) is the closest recovered peak within a half-line width distance and all corresponding pairs are mutually exclusive.
2. Accuracy of reconstructed frequencies: This is found by calculating the distance between the master and recovered peak positions (in Hz) for corresponding pairs of peaks (found as described above). Unlike the NUSCON metric, we only consider pairs where there is a clear correspondence between a master peak and recovered peaks so do not use the symmetric Hausdorff distance (i.e., the correspondence is only considered from master peaks to recovered peaks bringing the metric in line with intensities) and do not need to place an upper bound on the distances since peaks more than a half-linewidth apart would not be considered a pair.
3. False positive rate: this is the rate at which peaks are detected in the reconstructed spectrum that are not present in the master peak list, *i.e.*, the appearance of artefacts.
4. False negative rate: this is the rate at which peaks that appear in the original spectrum are not present in the reconstructed spectrum, *i.e.*, missing peaks.

The results of running this analysis 100 times each under different sampling rates (12.5% and 20% sampling corresponding to 32/256 and 51/256 complex points) and in the absence and presence of noise (gaussian noise with standard deviation 0.02) is summarised in Fig S2.

The results show that the FID-Net reconstruction tool is particularly useful when dealing with very sparse or noisy data. For example, at 12.5% sparsity with no added noise FID-Net produces very few false positive peaks compared to either the SMILE or hmsIST algorithms. Similarly, in the presence of noise, at both 12.5% and 20.0% sparsity hmsIST produces a relatively large number of false positives, while a number of true peaks cannot be clearly identified from the SMILE spectra. Conversely, the FID-Net reconstructions tend to find a better balance between minimising artefacts while maintaining recovery of actual peaks.

The weakness of the FID-Net reconstructions is that the recovered peak intensities and frequency accuracy is generally poorer than SMILE or hmsIST. This is particularly true for spectra with higher sampling (20%) and minimal noise spectra, where the reconstructions from both SMILE and hmsIST are superior. However, in the presence of noise the differences in intensity correlations and frequency deviations become much smaller, reflecting the strength of FID-Net reconstructions with noisier data. It should also be noted that where there is a higher false negative rate (peaks are not found in the reconstructed spectra) these are not included in the intensity correlations or frequency deviation calculations meaning these parameters may improve in the presence of greater noise.

Overall, this analysis suggests that the FID-Net method for reconstruction is particularly effective when dealing with very sparse or noisy data. On the other hand, at higher sampling rates and where the noise in the signal is minimal, while the performance of FID-Net is still reasonable, highlighting the flexibility of the approach, it is not as good as the SMILE or hmsIST algorithms for performing reconstructions.

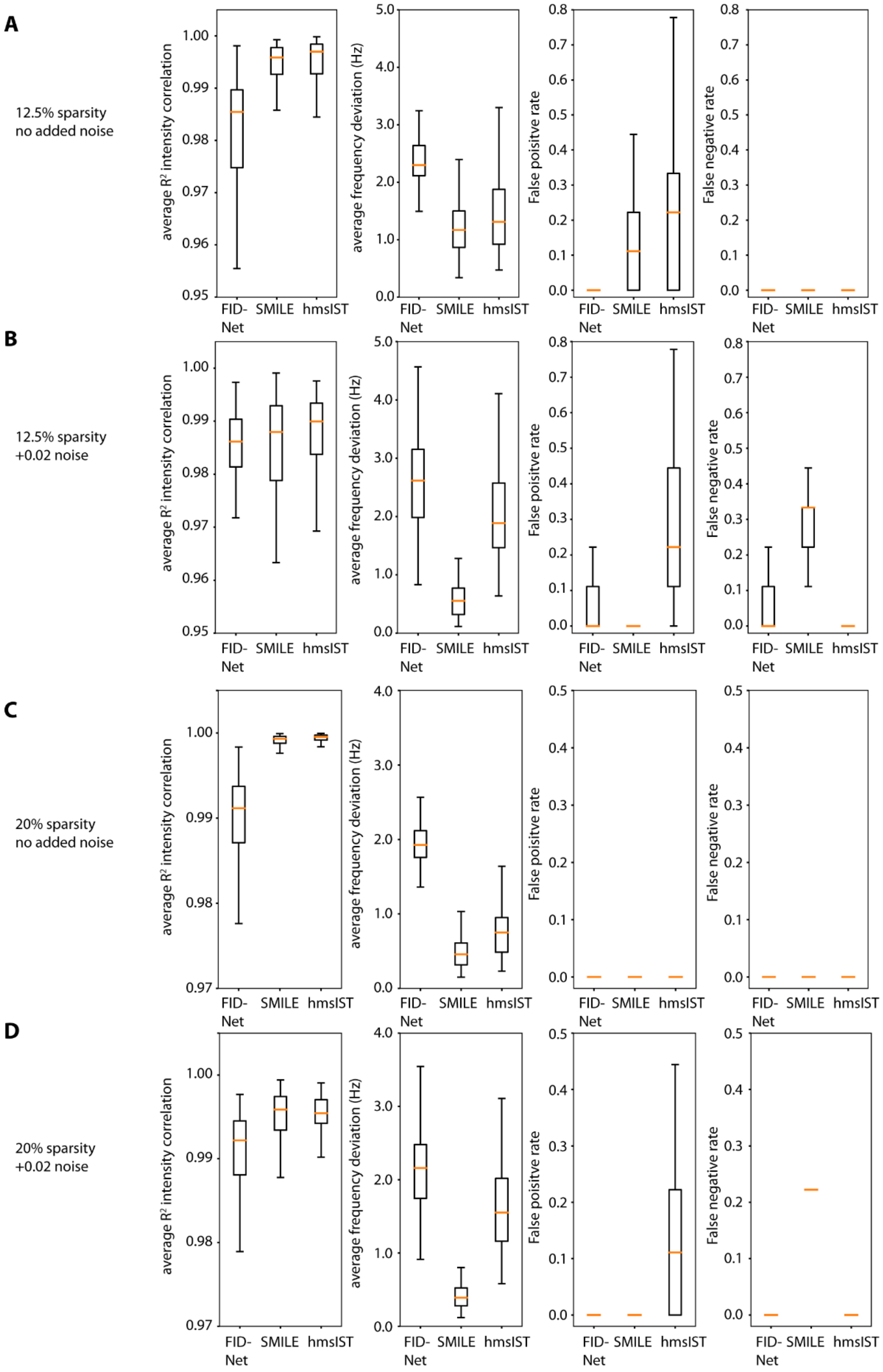

Fig S2 Box and whisker plots showing the performance of different algorithms in the reconstruction of synthetic NUS data (Fig S1) using NUSCON metrics. The reconstructions are carried out under conditions of **(A)** 12.5 % sparsity and no added noise **(B)** 12.5% sparsity and 0.02× max intensity added noise **(C)** 20.0% sparsity and no added noise and **(D)** 20.0% sparsity and 0.02× max intensity added noise. In the box and whisker plots the box indicates the interquartile range, the orange line indicates the median and the top and bottom whiskers extend to the last data point less than third quartile plus

1.5×inter quartile range and the first data point greater than the first quartile minus 1.5×inter quartile range.

Exemplar Synthetic Reconstructions

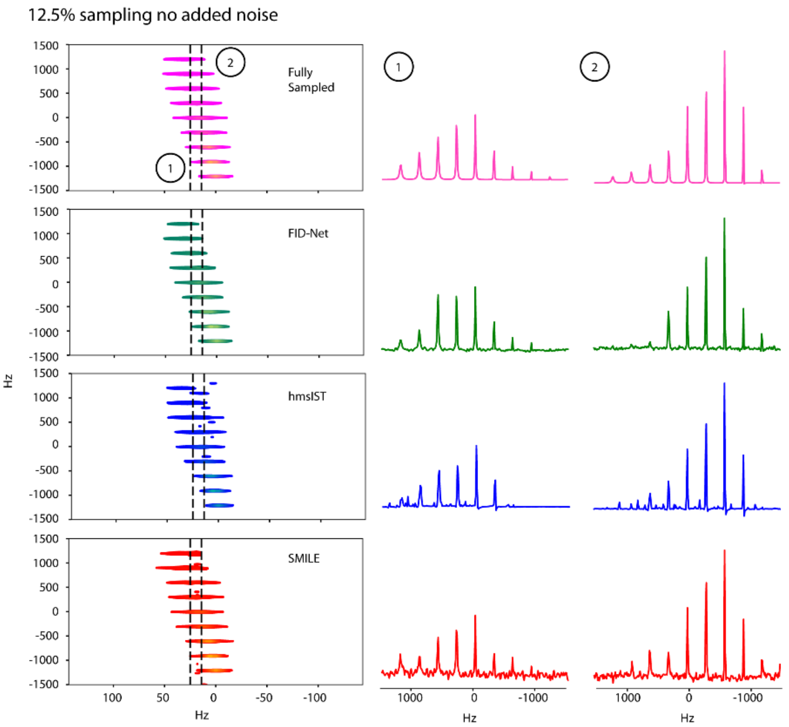

Fig S3 Exemplar reconstruction of synthetic spectrum using FID-Net, hmsIST and SMILE at 12.5% sparsity using a sinusoidally weighted Poisson-gap sampling schedule.

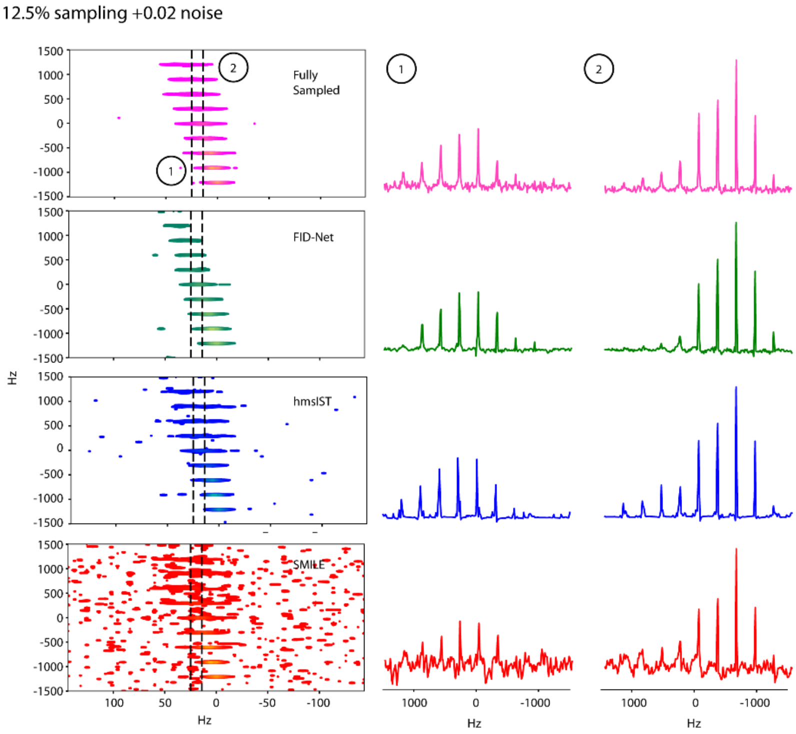

Fig S4 Exemplar reconstruction of synthetic spectrum using FID-Net, hmsIST and SMILE at 12.5% sparsity using a sinusoidally weighted Poisson-gap sampling schedule. Noise with 0.02× maximum signal is added to the spectrum.

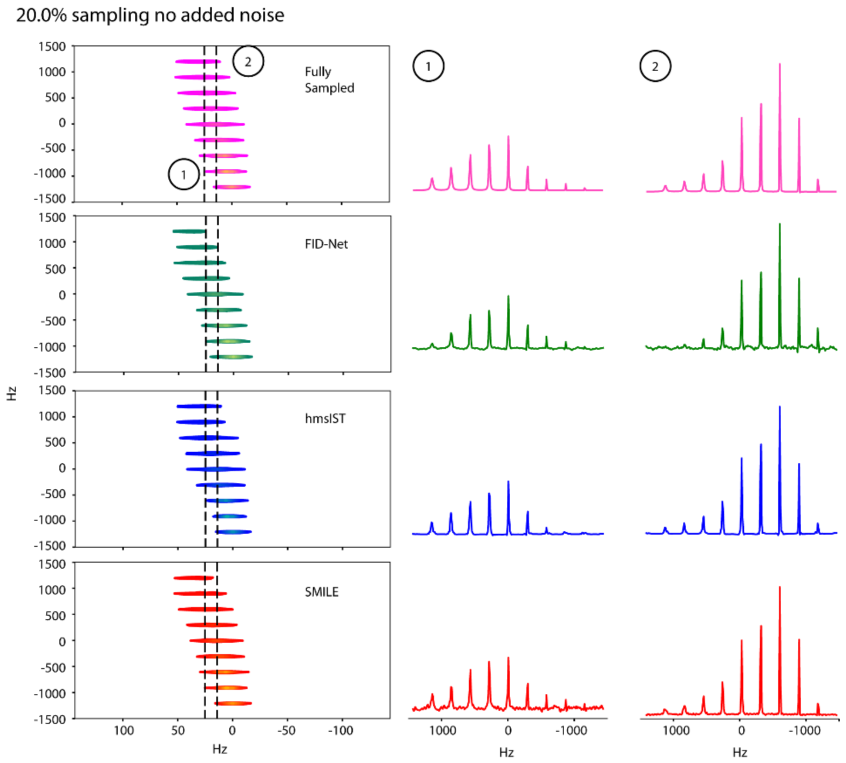

Fig S5 Exemplar reconstruction of synthetic spectrum using FID-Net, hmsIST and SMILE at 20.0% sparsity using a sinusoidally weighted Poisson-gap sampling schedule.

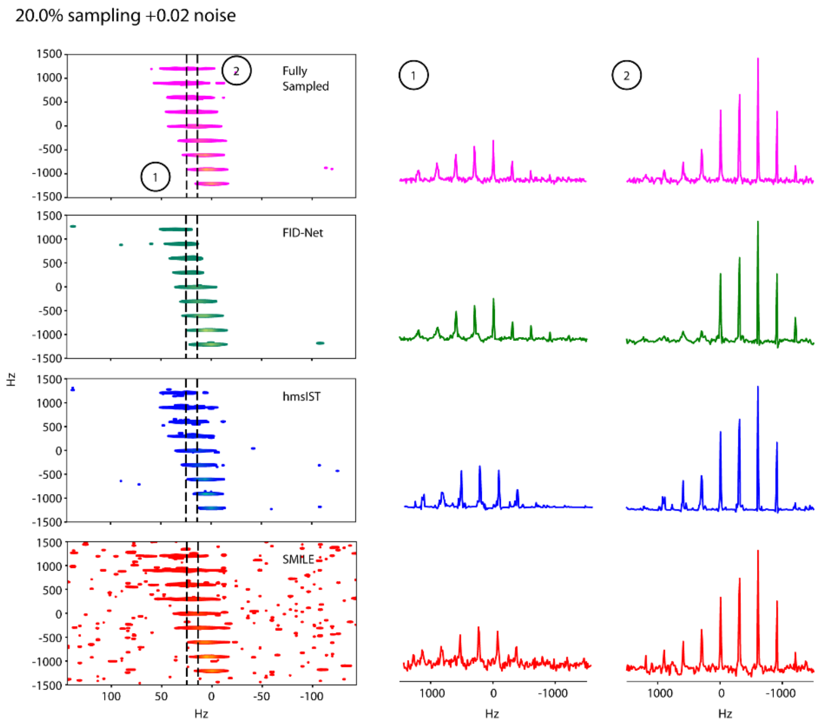

Fig S6 Exemplar reconstruction of synthetic spectrum using FID-Net, hmsIST and SMILE at 20.0% sparsity using a sinusoidally weighted Poisson-gap sampling schedule. Noise with 0.02× maximum signal is added to the spectrum.

Example Sampling Schedules:

SH3 Domain ABP1P<br>Fully sampled 120<br>complex points	T4 Lysozyme<br>Fully sampled 256<br>complex points	HDAC8<br>Fully sampled 192<br>complex points
0	0	0
1	1	1
2	3	2
4	4	4
5	5	6
7	8	7
11	12	9
17	14	11
23	16	17
27	20	21
39	24	27
49	28	31
68	34	37
87	39	46
114	45	59
	51	69
	58	79
	66	88
	71	105
	78	115
	87	130
	92	151
	100	174
	110	187
	129	
	136	
	155	
	172	
	194	
	210	
	233	
	249	

Table S1 Sampling schedules used for reconstructions in main-text Figure 3. These are all Poisson-gap with sinusoidal $0 \rightarrow \pi/2$ weighting sampling schemes with 12.5% sampling rate. Note these sampling schedules are used for the reconstructions that are explicitly shown in Fig 3 and bar chart statistics are based on reconstructions using 100 different Poisson-gap and random sampling schedules.

Examples of ‘Noisy’ Reconstructions

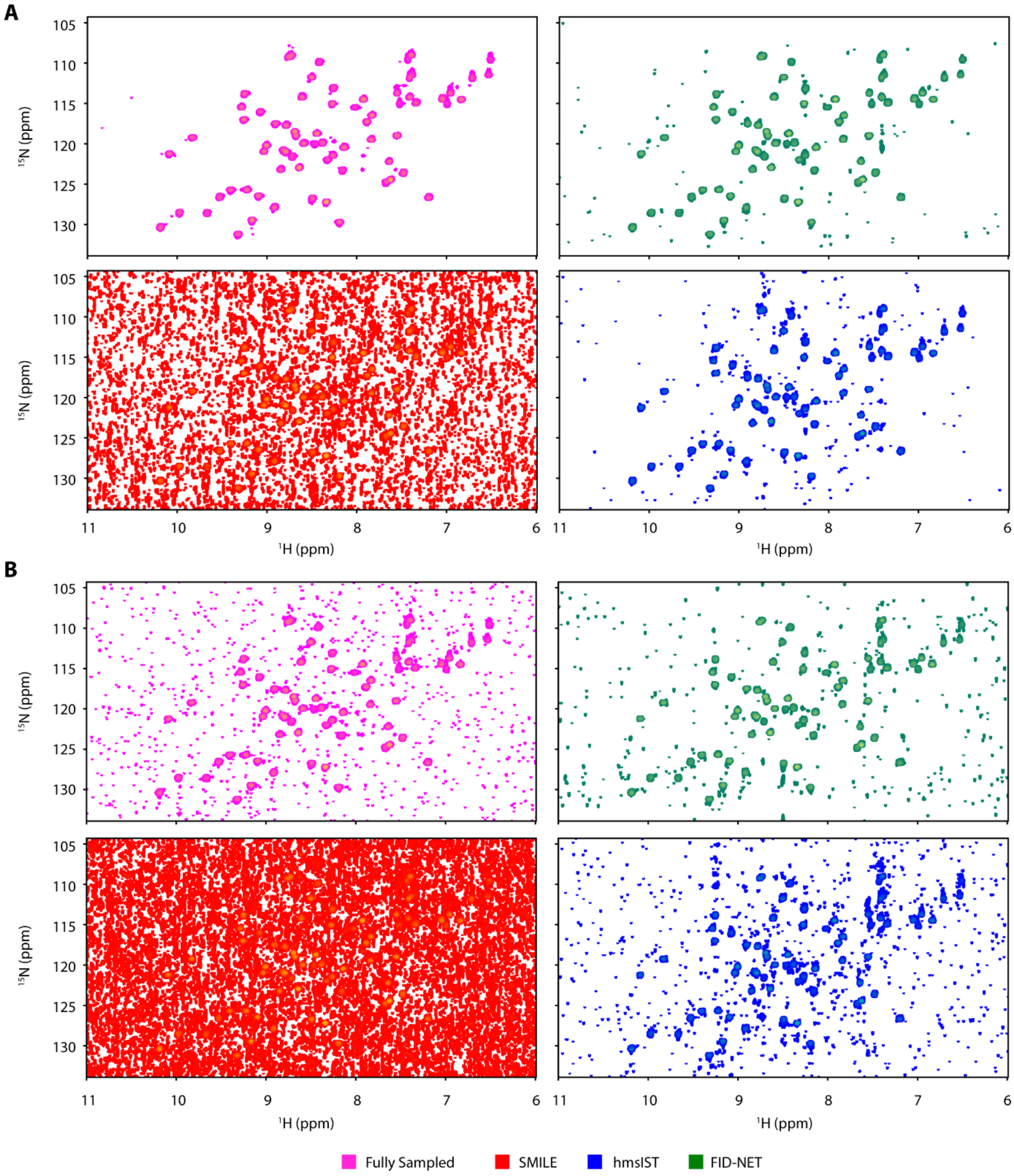

Fig S7 Exemplar reconstructions of ‘noisy’ sparsely sampled spectra of SH3 domain from ABP1P with SMILE, FID-Net and hmsIST. Gaussian noise with standard deviation equal to (**A**) 0.03× and (**B**) 0.05× the maximum intensity is added to the data prior to reconstruction. Reconstructions are performed at 12.5 % sampling and employ a sinusoidally weighted Poisson-gap sampling schedule. Only positive contours are shown.

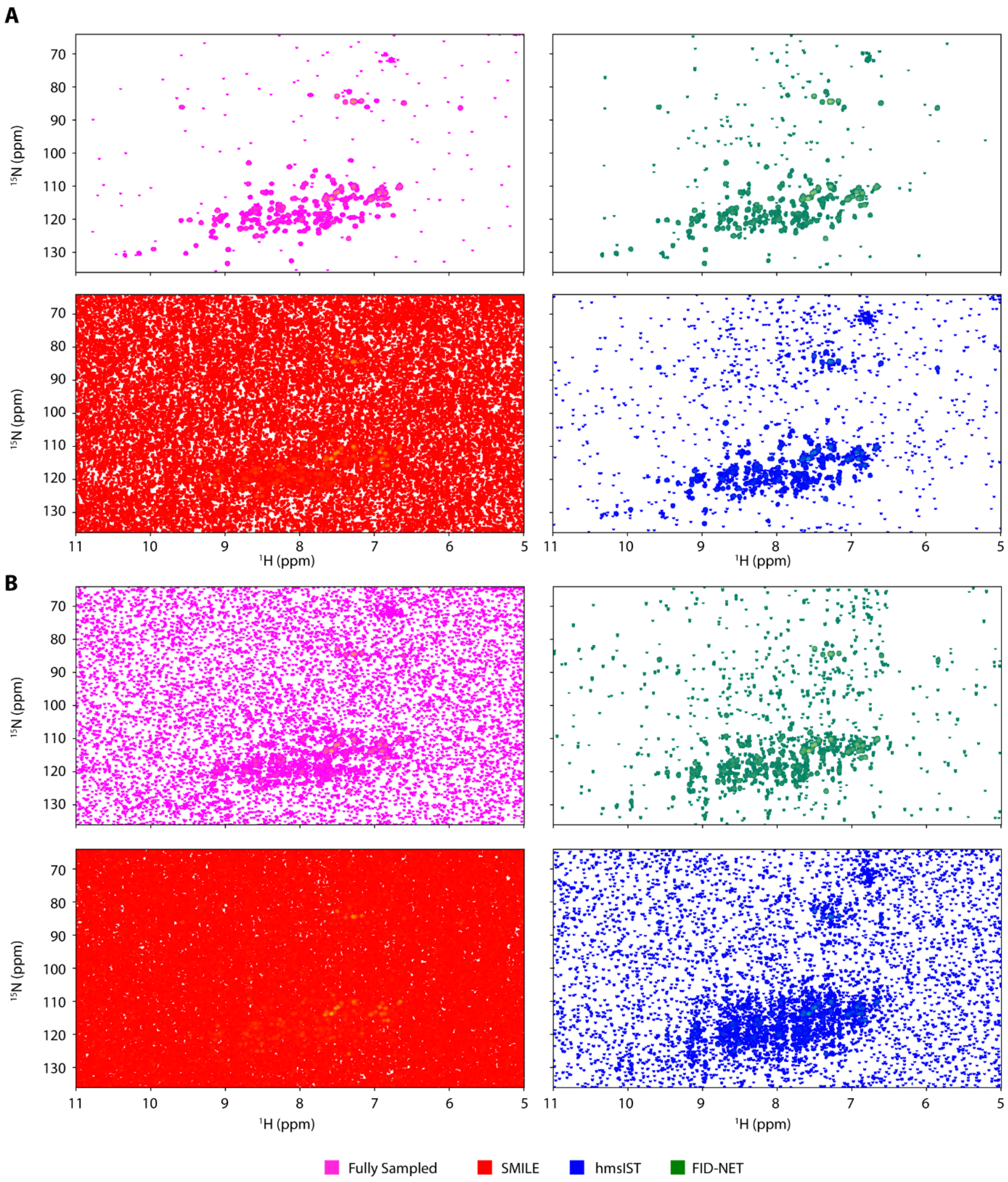

Fig S8 Exemplar reconstructions of 'noisy' sparsely sampled spectra of T4 Lysozyme with SMILE, FID-Net and hmsIST. Gaussian noise with standard deviation equal to (**A**) 0.03× and (**B**) 0.05× the maximum intensity is added to the data prior to reconstruction. Reconstructions are performed at 12.5 % sampling and employ a sinusoidally weighted Poisson-gap sampling schedule. Only positive contours are shown.

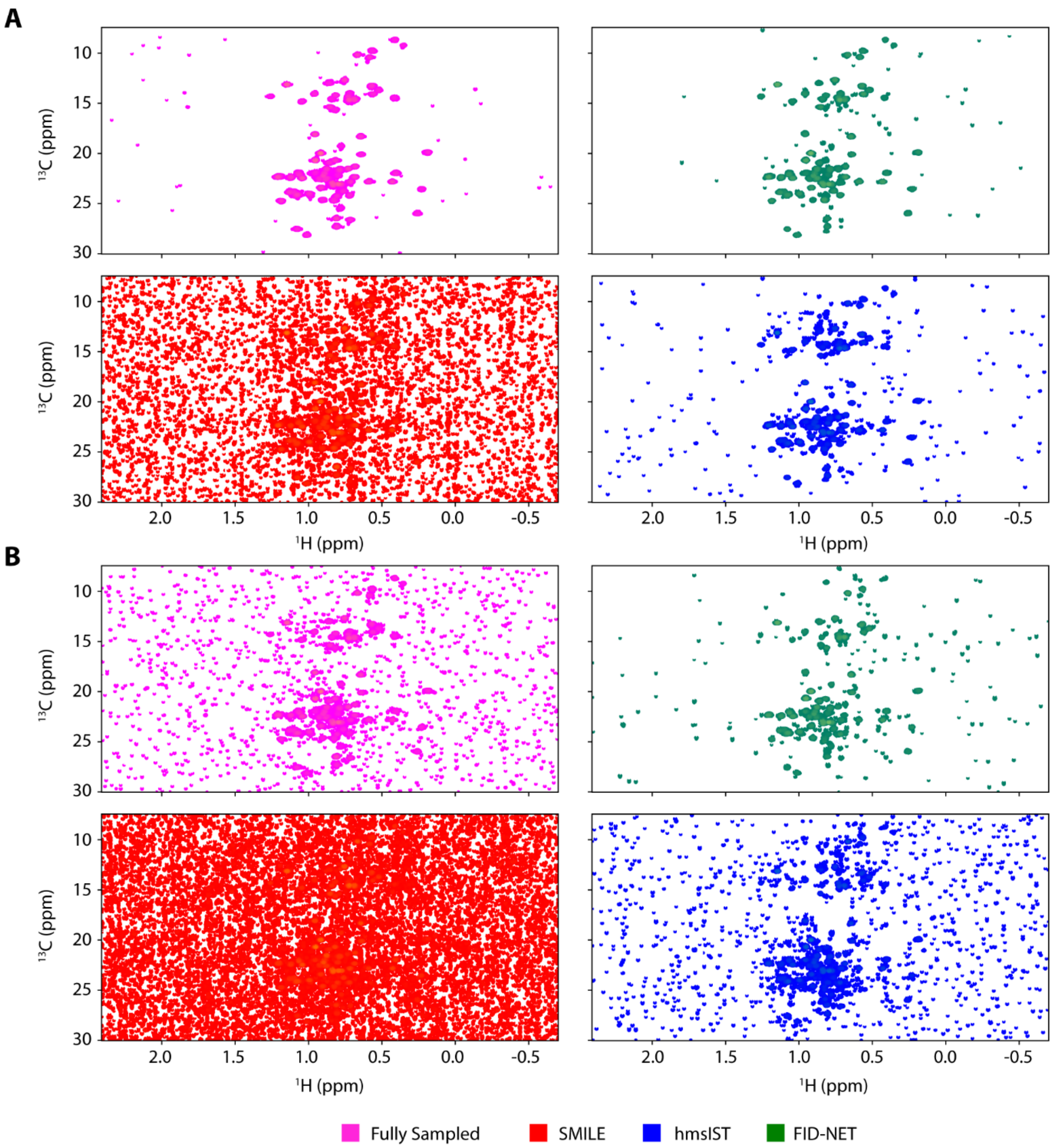

Fig S9 Exemplar reconstructions of 'noisy' sparsely sampled spectra of HDAC8 with SMILE, FID-Net and hmsIST. Gaussian noise with standard deviation equal to (**A**) 0.03× and (**B**) 0.05× the maximum intensity is added to the data prior to reconstruction. Reconstructions are performed at 12.5 % sampling and employ a sinusoidally weighted Poisson-gap sampling schedule. Only positive contours are shown.

Versatility of FID-Net with respect to sampling rate

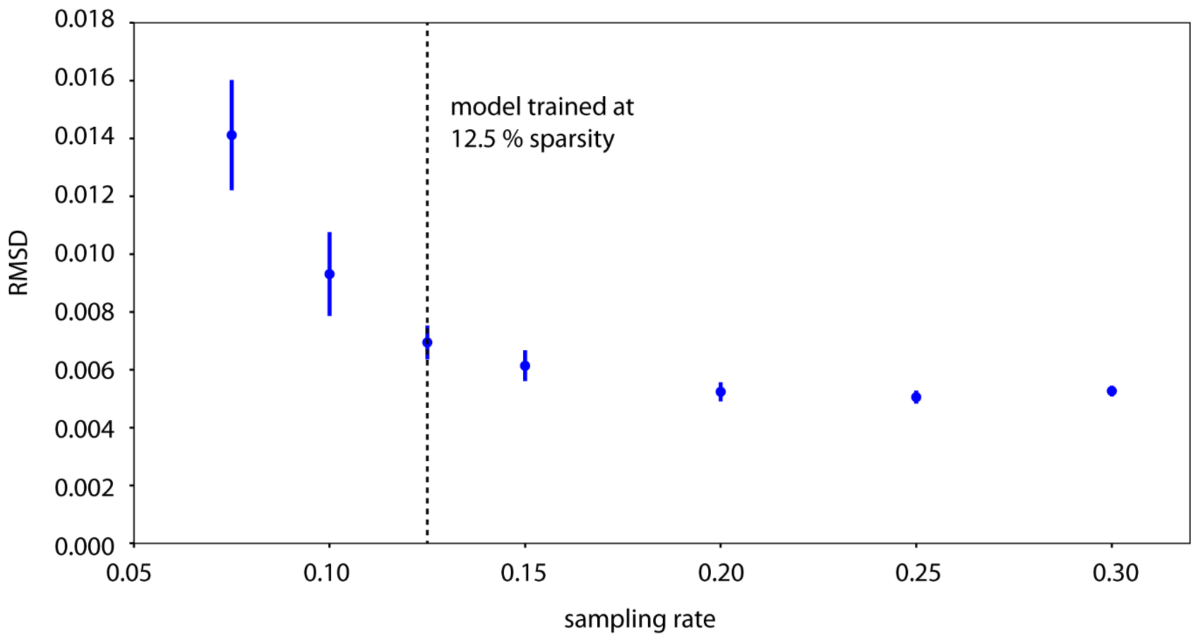

Fig S10 Variation of RMSD for FID-Net reconstructions of the SH3 domain ^{1}H-^{15}N HSQC (Fig 4A, main text) as a function of sampling rate. Each point represents the average RMSD from 100 Poisson-gap sampling trials at a given sampling rate and the vertical line indicates the standards deviation. As expected, when the sampling rate is reduced, there is a decline in the quality of the reconstructions. However, when the sampling rate is increased the quality of the reconstructions increases and plateaus at approximately 0.20, despite this being far from the sparsity the model is trained at.

Using Sliding Scale as a Metric for Uncertainty

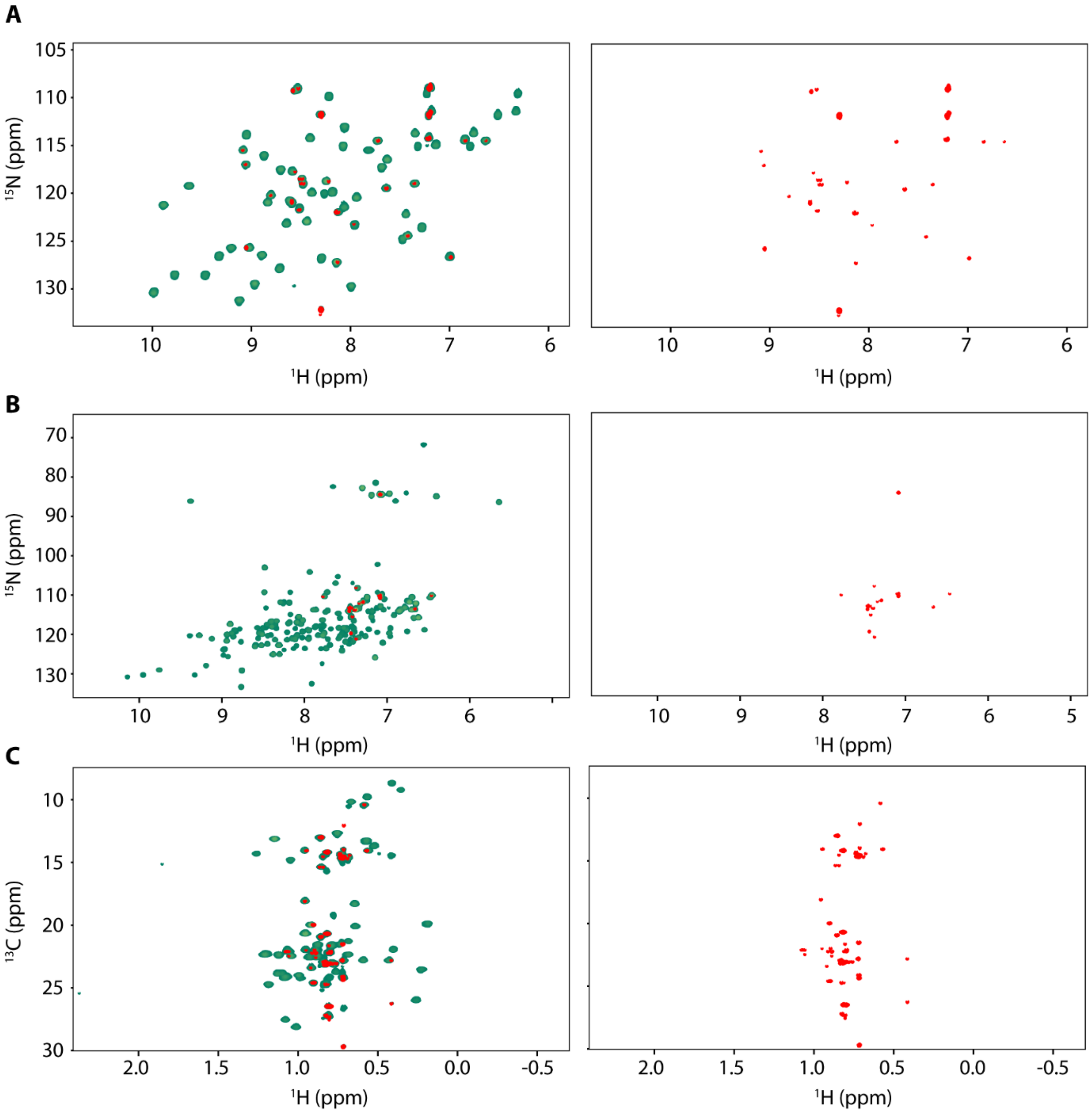

Fig S11 Plots of FID-Net reconstructions (green) using sampling schedules shown in Table S1 for the proteins (**A**) the SH3 domain from ABP1P, (**B**) T4 Lysozyme. and (**C**) human histone deacetylate 8 (HDAC8). The red plots show the standard deviations from the four reconstructions that are averaged to give the final reconstructed spectra. These are plotted on top of the spectra on the left and alone on the right. Where the two plots overlap this suggests an uncertainty in the peak intensity, linewidth or frequency or that the peak may be a false positive (artefact). Where there is uncertainty but no peak this suggests the possibility of a false negative at this position. As expected, more crowded vertical slices in the spectrum are generally associated with a higher uncertainty.

Python Code for Building FID-Net

```
import tensorflow as tf
from tensorflow import keras

def build_model_fidnet(num_blocks = 3, num_filters = 64):
    # note for reconstruction network num_filters = 64
    # and for decoupling network num_filters = 32
    def ft_layer(x):
        ft = tf.transpose(x, perm = [0,2,1,3])
        ft = tf.reshape(ft, [-1,4,256,2])
        ft = tf.complex(ft[:,:,:,0],ft[:,:,:,1])
        ft = keras.layers.Lambda(tf.signal.fft)(ft)
        ft = tf.transpose(ft, perm = [0,2,1])
        ft = tf.math.real(ft)
        return ft

    def waveLayer(x,num_filters,dil):
        y1 = keras.layers.Conv2D(filters = num_filters, kernel_size=[8,4],
                                 padding="same", dilation_rate=[dil,1])(x)

        y2 = keras.layers.Conv2D(filters = num_filters, kernel_size=[8,4],
                                 padding="same", dilation_rate=[dil,1])(x)

        y1 = keras.layers.Activation('tanh')(y1)
        y2 = keras.layers.Activation('sigmoid')(y2)

        z = y1*y2
        z = keras.layers.Conv2D(filters = num_filters*2,
                                kernel_size=[8,4], padding="same")(z)

        return keras.layers.Add()([z,x]), z

    dilations = [1,2,4,6,8,10,12,14,16,20,24,28,32]

    input =  keras.layers.Input(shape=[512, 4, 1])
    x = input
    skips = []

    for dil in dilations*num_blocks:
        x, skip = waveLayer(x, num_filters, dil)
        skips.append(skip)

    x = keras.layers.Activation("relu")(keras.layers.Add()(skips))
    x = keras.layers.Conv2D(num_filters, kernel_size=[8,4],
                            padding="same", activation="relu")(x)
    fin = keras.layers.Conv2D(1, kernel_size=[8,4],
                              padding="same", activation="tanh")(x)
    ft_fin = ft_layer(fin)
    model = keras.Model(inputs=[input], outputs=[fin,ft_fin])
    model.compile(loss=["mse","mse"], loss_weights = [0.0,1.0],
                  optimizer=keras.optimizers.RMSprop(lr=1.0e-4))

    return model
```

Supplementary References

[1] J. Ying, F. Delaglio, D. A. Torchia, A. Bax, *J. Biomol. NMR* **2017**, *68*, 101–118.

[2] S. G. Hyberts, A. G. Milbradt, A. B. Wagner, H. Arthanari, G. Wagner, *J. Biomol. NMR* **2012**, *52*, 315–327.

[3] F. Delaglio, S. Grzesiek, G. W. Vuister, G. Zhu, J. Pfeifer, A. Bax, *J. Biomol. NMR* **1995**, *6*, 277–293.
